# Searching Dynamic Agents with a Team of Mobile Robots

**DOI:** 10.3390/s120708815

**Published:** 2012-06-27

**Authors:** Miguel Juliá, Arturo Gil, Oscar Reinoso

**Affiliations:** Systems Engineering and Automation Department, Miguel Hernandez University of Elche, Avda. Universidad s/n. Edif. Quorum V, 03202 Elche, Alicante, Spain; E-Mails: arturo.gil@umh.es (A.G.); o.reinoso@umh.es (O.R.)

**Keywords:** dynamic agent search, grid-based Bayesian filtering, search algorithm, multi-robot systems

## Abstract

This paper presents a new algorithm that allows a team of robots to cooperatively search for a set of moving targets. An estimation of the areas of the environment that are more likely to hold a target agent is obtained using a grid-based Bayesian filter. The robot sensor readings and the maximum speed of the moving targets are used in order to update the grid. This representation is used in a search algorithm that commands the robots to those areas that are more likely to present target agents. This algorithm splits the environment in a tree of connected regions using dynamic programming. This tree is used in order to decide the destination for each robot in a coordinated manner. The algorithm has been successfully tested in known and unknown environments showing the validity of the approach.

## 1. Introduction

This paper investigates the search problem in which a team of agents (the searchers) collectively try to find another set of moving agents (dynamic targets). The interest in this particular problem stems from the fact that, in the last years, a great number of applications have emerged in which it is necessary to deploy search tasks in unstructured environments. For example, search and rescue tasks, surveillance and other military tasks. The deployment of mobile robots in these situations is advantageous, since it avoids the presence of humans in dangerous places or in environments difficult to reach. In addition, mobile robots can take advantage from the use of sensors specially designed for the detection of objectives, such as motion detectors, infra-red cameras or laser range finders.

The dynamic agents search problem is related to the pursuit and evasion problem [1,2]. The pursuit and evasion problem describes a two teams game in which the agents in the Pursuer Team try to find the agents of the Evader Team. Since the agents of the Evader Team are in motion, the problem is not reduced to a simple exploration problem as it would be if they were static. In this case, the pursuers should make a fast coverage of the environment trying to find all the evaders. However, since the evaders may enter an area previously covered by the pursuers, the pursuers may cover completely the environment without finding all the evaders. As a consequence, a simple exploration algorithm [3] is not enough to perform this task. Consequently, most of the pursuit and evasion techniques try to sweep the environment with the team in such an optimal way that there is no possible escape for the evaders. That is, provided that there are enough pursuers, an optimal path can be found for each pursuer in order to cover all the search area without letting any opportunities for the evaders to escape. This kind of algorithms fail in the case there are not enough pursuers for a given scenario. In this sense, when not enough searchers are provided more general approaches are necessary.

We focus in this paper on the dynamic agents search problem, which is the particular case of the pursuit and evasion problem in which the evaders are not intelligent and their movements do not try to avoid the pursuers. This particular case of searching a moving agent that is not trying to escape is quite common in robotics applications. For instance, a robot with human-robot interaction capabilities may need to find a human in its proximities in order to interact with and get some information [4]. Other application scenario is the case of searching for malfunctioning robots that may be moving around without control.

The proposed problem will be approached in a general way, assuming there are not enough robots to perform a full optimal search of the target area. In this sense, the robots choose to explore first those areas where it is more likely to find the moving targets. Next, the main hypotheses that are considered in this approach are detailed. Firstly, it is assumed that the searchers know the top speed of the dynamic agents, so the movement of these moving targets can be delimited. In the majority of the applications, it can always be assumed that a maximum speed for the dynamic agents can be determined. Secondly, it is assumed that the searchers are equipped with an appropriate sensory system that is able to detect each one of the targets within a range. Nowadays, the use of omnidirectional vision systems allows to assume this premise. In addition, the team of searchers consists of a set of mobile agents that maintains a communication channel around all the environment. Besides, the problem of the localization and reconnaissance is obviated.

Consequently, provided that the searchers know the top speed of the targets, the movements of the targets can be completely delimited in a probabilistic way. Therefore, from this information and from their own trajectories and sensor readings, the Search Team can make an estimation of the areas of the environment that are more likely to hold a target. In order to integrate all this information a grid-based Bayesian filter (GBBF) is used. Then, a navigation algorithm selects the areas according to the GBBF where targets are more likely to be present as destinations for the searchers. In order to avoid that all the searchers go to the same place, some coordination mechanisms are applied in the destination selection.

The algorithm presented here has been tested in simulation in two different cases. First, we consider that the search team knows precisely the environment, however the whole environment needs to be explored in search of a set of moving targets. Second, we consider that the environment is completely unknown. In this case, a map of the environment is built as the search team covers the different areas of the environment. The map being built is used in order to improve the search of the dynamic agents.

The rest of the paper is organized as follows. First, in the next section we present some work in close relation with this paper. Second, the grid-based Bayesian filter used to estimate the best target areas is explained in Section 3. Next, the coordinated goal selection for each agent of the Search Team is developed in Section 4. Then the experiments carried out to test the approach are exposed in Section 5. Finally, Section 6 explains the conclusions and open research lines.

## 2. Related Work

In mobile robotics, exploration is considered as the problem of traversing all the areas of a particular unknown environment [3]. Normally, exploration algorithms are used jointly with a Simultaneous Localization and Mapping (SLAM) algorithm in order to create a map of the environment during the exploration process [5]. Classical exploration techniques direct the robots to examine every place in the environment in an efficient manner, thus reducing the total time needed to cover a determined area. For instance, some algorithms command the robots to the nearest frontier [6]. In these techniques, the environment is represented in a discrete bi-dimensional grid, where each cell is considered as occupied, free or unknown (unexplored). Frontier cells are unknown cells that lie next to a free cell. The application of this family of algorithms to search problems is direct [7].

When the main objective is to incrementally build a map of the environment, exploration strategies allow to select the vantage points that should be reached by the robots. In these activities, it is of capital importance to design a good exploration strategy in order to build the most precise map in the shortest time. Most of the exploration algorithms presented to date consider the evaluation of an utility function in order to select the best trajectory for the robot [8]. Some authors have extended these ideas to the case of multi-robot exploration. Commonly, the utility function used in exploration is a measure of the information gain of visiting a place in the environment and the cost of reaching it. The cost is in relation with the distance between the robot and the possible destination, whereas the utility is estimated in dissimilar manners. For example, in [9] utility is considered as the visible area behind a frontier. In [10], the destinations chosen by other robots are taken into account in the computation of the utility function. Thus, when a robot is commanded to a point in the environment, the utility of this area is reduced for the rest of the robots, in an effort to coordinate them and increase the exploration speed. A market-based mechanism where the robots compete and optimize their routes by negotiating their destinations as a function of the cost and the expected utility was suggested in [11]. Some other approaches focus on the structure of the environment. For example, the doors found in the environment can be recognised and represented inside a topological map. In [12], this information is considered in order to assign utility values to different unexplored areas for each robot.

This kind of search and exploration algorithms has been applied to search and rescue tasks. In these situations, we typically wish to search and localize a series of targets or victims inside the search area, being the number of objectives to localize a priori unknown. The approaches in this field normally have to deal with the problem of accessing and manoeuvring in these environments [13], thus in search and rescue applications researchers are generally more concerned with the development of algorithms that allow the robot to manoeuvre in completely unstructured and challenging environments. In consequence, the processing of sensor information is of paramount importance in order to find the free navigable space and obstacles. In addition, the localization of the targets inside this environments may be extremely complicated [14]. There are different variations in the search and rescue problems used for the detection of a set of static targets. In these cases, and since there exists no prior knowledge about the position of the targets, the search and exploration problem can be stated as the maximization of the number of detected targets in a fixed amount of time, or equally, the maximization of the explored area. For example, an approach based on a Multi Criteria Decision Making algorithm that allows to define the exploration strategies deployed by a set of robots in a search and rescue problem is presented in [15]. Other exploration strategies try not only to maximize the explored areas, but to find and communicate the position of the victims. Thus, in [16], the exploration scheme is based on a utility function computed with several criteria that consider the distance, the expected information gain and the probability of a successful communication from the candidate destination.

Another applications that requires the development of search and exploration algorithms is the pursuit and evasion problem, in which a set of agents try to chase and localize a number of intruders [17]. The main difference with respect to the before mentioned problems is that, in this case, the targets try to run away from the searching robot. Thus, in these situations, the objective of the evader is to stay out of the reach of the pursuer, whereas the main task of the pursuer is to localize and capture the evader. In many cases, the position of the targets and the searchers is assumed to be known at each time, thus leading to an optimization problem [18]. For instance, in [1], an environment in which both the evaders and the pursuers have knowledge over the position of their opponents is considered.

In other cases the position of the evaders is unknown. For instance, in [2] the objective is to localize a set of moving targets by making use of a prediction scheme. Since the movement of the evader is unknown by the pursuer, the robots have to make predictions based upon the history of movements performed by the evader. In this sense, an intelligent search method in which a subset of the pursuers (the blockers) is positioned at some strategic points in the environment with the purpose to avoid the free movement of the evaders was presented in [19]. A novel visibility-based pursuit and evasion problem where a searching team needs to coordinate to find the evaders in a bi-dimensional environment was addressed in [20].

A particular application of the pursuit and evasion problem with unknown evaders positions consists in the surveillance and supervision of buildings, industrial estates and other restricted areas [21]. In this kind of task it is crucial to explore periodically the environment in order to verify the non-existence of intruders. For instance, some authors have used a subdivision the environment as a set of polygonal regions [22].

Little attention have been paid to the intermediate problem in which the target agents are in motion but they do not try to deliberately escape. As we said, this could be the case of robots with human-robot interaction capabilities seeking for humans in order to interact with, or the case of looking for malfunctioning robots moving around. Pursuit and evasion techniques could be applied to these cases. However, when the environment is complex a great number of robots may be necessary in order to perform an optimal search. Consequently, a new technique specifically designed for this case has been developed and it is exposed in this paper.

## 3. Grid-Based Bayesian Filter

The Search Team needs to track the positions that are more likely to hold a target. This is done by means of a GBBF. The search area *C* is divided using a grid. The total number of cells in *C* will be denoted by *T*. Each cell *c* of the grid has a probability *P*(*c_k_*) of holding a target in the time step *k*. This probability will be evaluated for a single target. Since the multiple targets case would have the same probability to that of the single target case multiplied by a constant, it does not affect the planning. In this sense the algorithm works independently of the number of targets. Next, the initialization of the map and the prediction and update of the probabilities in the subsequent steps are detailed.

### 3.1. Initialization

In the beginning, it is assumed that all cells have the same probability of holding a target agent:

(1) $$P\left(c_{0}\right)=1/T,\forall c\in C$$

This initial probability *P*(*c*_0_) needs to be modified in time. On the one hand, since the targets are in motion, there exists a possibility for the dynamic targets to enter an area previously visited by the searchers. In this sense, the probability of each cell needs to be propagated to its neighbours periodically as a function of the speed of the targets and the resolution of the grid. This change in the probability will be evaluated in the prediction stage. On the other hand, the actions of the searchers with their observations modify the probabilities in the grid too. This change in the probability is a consequence of the reduction of the area where the targets can be found and it will be covered in the update stage. The observations consist of the different subsets of cells *Z*_0:_*_k_* = {*Z*_0_, *Z*_1_, …*Z_k_*}, with *Z_i_* ∈ *C*, that have been found to be clear at each time step. In the following, in order to denote that the cells are clear according to the observation, the terminology *Z̄* will be used. In this sense, *P*(*Z̄*) expresses the probability that no targets are found in the subset of cells *Z*, that is, *P*(*Z̄*) = Π*_z_*_∈_*_Z_ P*(*z* = *False*). The prediction and update stages are described below.

### 3.2. Prediction Stage

The prediction stage consists in the propagation of the probability of each cell in the grid to its neighbours according to the resolution and the target top speed. For instance, a maximum linear speed of 0.3 *m/s* and a resolution of 0.1 *m* can be used with a 3 *Hz* probability prediction frequency. Since no other information about the movement of the targets is known by the searchers apart from their top speed, in order to model the motion of the targets it is assumed that they can stay in their current cell or move to one of the contiguous cells (when they are not occupied) with the same probability. The next equation models the result of the prediction stage:

(2) $$P\left(c_{k}|{\bar{Z}}_{0:k-1}\right)=\sum_{n\in N_{c}}P\left(c_{k}|n_{k-1},{\bar{Z}}_{0:k-1}\right)P\left(n_{k-1}|{\bar{Z}}_{0:k-1}\right)$$

where *N_c_* is the 3 × 3 subset of cells centred on *c*. The term *P*(*n_k_*_−1_∣*Z̄*_0:_*_k_*_−1_) is the prior, and the term *P*(*c_k_*∣*n_k_*_−1_, *Z̄*_0:_*_k_*_−1_) models the probability of moving from one of the cells in *N_c_* to *c*. As the robots move, another grid is created with occupancy information obtained with range finder sensors in the robots [23]. The information from the occupancy grid is used to obtain the transition probabilities. As we said, it is assumed that a moving target can stay in their current cell or move to any of the contiguous cells when they are not occupied with the same probability.

### 3.3. Update Stage

For each time step *k*, each searcher sweeps a new area with its sensors. The subset of cells *Z_k_* will denote the total area swept by all the Search Team adding all the individual areas. Furthermore, if the map is previously unknown and the searchers are simultaneously building it, it is likely that with the addition of new obstacles to the grid map some area appears to be closed and therefore inaccessible. Therefore *Z_k_* includes three kinds of cells: obstacles detected, non-accessible cells detected, and non-occupied cells. Since the sensing of these areas reduces the area where the targets can be found, the probability to find a target in the other zones increases. This growth can be updated in the grid applying Bayes' rule:

(3) $$P\left(c_{k}|{\bar{Z}}_{0:k}\right)=\frac{P\left({\bar{Z}}_{k}|c_{k},{\bar{Z}}_{0:k-1}\right)P\left(c_{k}|{\bar{Z}}_{0:k-1}\right)}{P\left({\bar{Z}}_{k}|{\bar{Z}}_{0:k-1}\right)}$$

where, *P*(*Z̄_k_*∣*c_k_, Z̄*_0:_*_k_*_−1_) = 1, ∀*c_k_* ∉ *Z_k_* and *P*(*Z̄_k_*∣*c_k_, Z̄*_0:_*_k_*_−1_) = 0, ∀*c_k_* ∈ *Z_k_*. Furthermore, *P*(*Z̄_k_*∣*Z̄*_0:_*_k_*_−1_) can be expressed in terms of its complementary *P*(*Z̄_k_*∣*Z̄*_0:_*_k_*_−1_) = 1 − *P*(*Z_k_*∣*Z̄*_0:_*_k_*_−1_). Therefore, we have that

(4) $$P\left(c_{k}|{\bar{Z}}_{0:k}\right)=\{\begin{array}{ll} \frac{P\left(c_{k}|{\bar{Z}}_{0:k-1}\right)}{1-\sum_{z\in Z}P\left(z_{k}|{\bar{Z}}_{0:k-1}\right)}, & \forall c_{k}\notin Z_{k}\\ 0, & \forall c_{k}\in Z_{k} \end{array}$$

where all terms can be found from the prediction stage.

To summarize the creation of the target probability grid, Equation (1) is used to initialize the map, Equation (2) is used for each time step *k* in order to propagate the probabilities according to feasible movements of the targets and Equation (4) is employed in order to update the map with the new information acquired by the searchers respectively.

**Figure 1.**
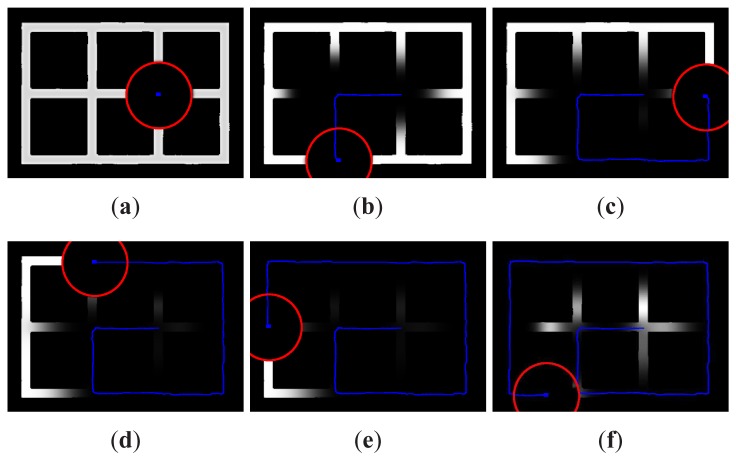
Evolution of the target probability map. The grey level indicates the normalized probability of each cell, corresponding the dark zones to low probability and the light zones to high probability. The red circles indicate the range of the sensor and the blue lines show the trajectory of the robot.

In order to clarify how the target probability mapper tracks the most likely areas to hold a target, Figure 1 has been included. This figure shows the evolution in time of the target probability map created by the GBBF for a search scenario with only one searcher. In the figure, the probability of each cell has been normalized with the maximum value for each time step. In this way, dark areas correspond to low probability, whereas light zones correspond to high probability. The red circles indicate the range of the sensor and the blue lines show the trajectory of the robot. This example assumes that the robot has initially a map of the environment. Therefore, all the obstacles are included in the first update observation *Z*_0_ and they appear always in black. In step 1 (Figure 1(a)), all cells have the same probability of holding targets except the non-accessible zones and the area initially covered by the sensors of the searcher. As the searcher moves in seek of the targets, more areas are obscured (Figure 1(b) and 1(c)). However, since the targets are in motion, the propagation stage inserts some probability for the targets to be in areas previously covered by the searcher. This effect can be clearly seen in the central areas of the Figure 1(d) and 1(e). In the end (Figure 1(f)), the searcher completes the exploration of all the environment. Despite the fact that all the areas have been covered, it can be seen how specially those areas that have not been covered for a long time appear to be the most probable areas to hold a target now.

## 4. Search Algorithm

As it has been seen, Section 3 describes how the target probability map is built. This map jointly with an occupancy grid map is used to decide the movements of the searchers in order to find the targets. Whereas the construction of the maps is made in a centralized way by all the searchers, the navigation planning is distributed. In this subsection, the process that each searcher follows in order to decide the next goal for its navigation is explained. Once the goal has been determined, a simple low level navigation function is used to give appropriate control actions to reach the goal.

Firstly, a tree decomposition of the environment using dynamic programming similar to the exposed in [24] is applied. In this tree *T*〈*nodes, edges*〉, each node *N_i_*(*t_i_, c_i_, ρ_i_, R_i_, γ_i_*) of type *t_i_* represents a position *c_i_* in the environment, the total cost to arrive to that node *ρ_i_*, a region of cells associated *R_i_*, and the number of searchers *γ_i_* in that region *R_i_*. There are three types of nodes: the root node (*t^r^*), gateway nodes (*t^g^*) and frontier nodes (*t^f^*). Edges *E_l,m_*(*d_l,m_, N_l_, N_m_*) represent the straight line path connecting the respective nodes *N_l_* and *N_m_* requiring to travel a distance *d_l,m_*. Next, it is explained how to create this tree.

Initially, the root node *N*_0_(*t*_0_, *c*_0_, *ρ*_0_, *R*_0_, *γ*_0_) of the tree corresponds to a node of type root *t*_0_ = *t^r^* with the current position *c*_0_ of the agent, a cost required *ρ*_0_ = 0 and a region *R*_0_ that is the subset of visible cells *S*_0_ in the range of the sensor with a number of searchers inside that subset *γ*_0_. The subset *R*_0_ = *S*_0_ is also used to initialize a processed cells mask *M* = *R*_0_. As it is shown in [24], gateway cells can be defined as the cells in the boundary of the visible area *S*_0_ that are contiguous to free cells not belonging to *S*_0_. These cells can be grouped to obtain chains of contiguous gateways cells. A new node *N_b_* of type gateway *t_b_* = *t^g^* and an edge *E*_0,*b*_ is added for each chain *b* of gateway cells found. The positions *c_b_* for the new nodes are situated in the cells in the middle of each chain. All these new nodes are also added to a list of unprocessed nodes. If the map was not known a priori, it might also be frontier cells in the region *R*_0_. Frontier cells are defined as free cells contiguous to unknown cells. In the same way, they are grouped in chains, and a new node of type frontier *t^f^* with its corresponding edge is added to the tree and to the list of unprocessed nodes for each chain of frontier cells.

**Figure 2.**
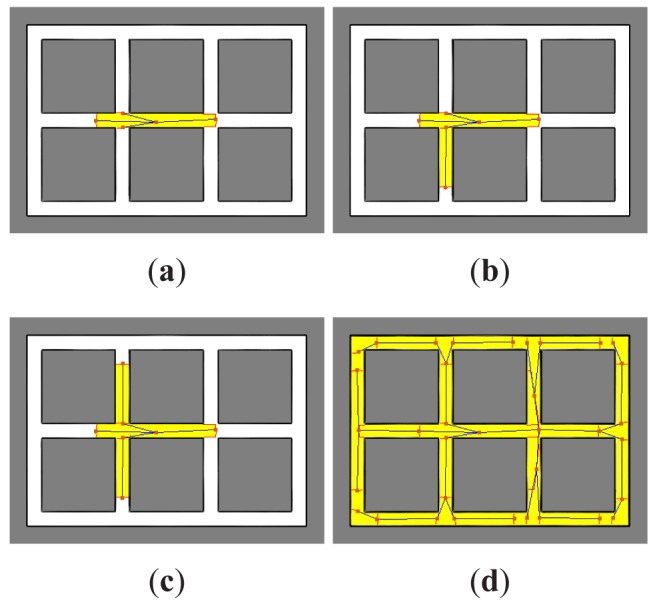
Tree segmentation of the environment.

Figure 2 shows the tree creation process for one searcher in a known environment. In Figure 2(a), it can be seen how the root node and the first level nodes (represented by squares) have been added to the tree, one per each chain of gateways cells found. The processed zone has been emphasized in the example. Next, the node *N_i_* from the list of unprocessed nodes that requires to travel the shortest distance *ρ_i_* is selected to expand the tree and subtracted from the list, being *ρ_i_* the sum of all the costs *d_l,m_* from the root node *N*_0_ to the *N_i_* node. The region *R_i_* associated to the node *N_i_* is established in different ways in function of the type of node. For a gateway node, *R_i_* is the subset *S_i_* of expected visible cells from the position *c_i_* excluding the cells in the mask and only those cells that are free. That is, *R_i_* = *S_i_* ∩ *M*^∁^ ∩ *F*, being *F* the subset of free cells. Next, the non-connected cells are also removed. For a frontier node, *R_i_* is the subset of expected visible cells *S_i_* but only those cells that are unknown. That is, *R_i_* = *S_i_* ∩ *U*, being *U* the subset of unknown cells. In the case of a gateway type node, new gateway cells and frontier cells can be identified in the region *S_i_*. However, in order to expand the tree over the environment not including branches that return to processed zones, the gateway cells are filtered with the mask *M*, and gateways not connected through the associated region *R_i_* are also removed. The corresponding new nodes and edges are added to the tree and to the unprocessed list and the mask is updated *M* = *M* ∪ *R_i_*. Figure 2(b) shows how the tree have been expanded. As it can be seen in Figure 2(c), the process continues with the lower cost node in the list. And it is repeated until all the nodes have been processed (Figure 2(d)).

Once each searcher has built its own tree, it has to be evaluated in order to be able to decide the next goals. In this sense, the objective of this evaluation of the tree is deciding a destination and a sequence of nodes to be used as way points for the next movements to reach the destination.

Firstly, since each node has a cost *ρ_i_* and some utility regarding the probability of finding a target in its associated region *P*(*R_i_*), a profit function *B*(*N_i_*) can be established to measure the importance of visiting each node *N_i_*:

(5) $$B\left(N_{i}\right)=\frac{P\left(R_{i}\right)}{\rho_{i}}$$

where the *P*(*R_i_*) = Σ*_c_*_∈_*_R_i__ P*(*c*) is the sum of the probabilities of the cells in that region *R_i_*. Using these profit values, a simple choice for one robot would be selecting the node of maximum profit as destination and the intermediate nodes as way points. However, since each searcher is part of a team, it has to consider positions of the other members of the team in order to cooperate. Furthermore, it has to consider also the profit of the intermediate nodes.

In this sense, the next recursive function *V*(*N_i_*) gives each node *N_i_* a value that takes into account its profit and the one from the best child node when this profit is higher than its own profit. In this case of finding a child node with higher profit, this node is marked as the next in the path for that branch in order to rebuild later a full path. It could be considered the best child node always. However, since the estimation of the probabilities and the position of the other agents changes often, it is better not to do too long term planning. Thus, the algorithms stops considering child nodes if they do not provide a significant profit. Besides, the function penalizes the nodes where other searchers *γ_i_* are present in the area associated to the node:

(6) $$V\left(N_{i}\right)=\{\begin{array}{ll} \frac{B\left(N_{i}\right)+\max_{j}V\left(N_{j}\right)}{{\left(1+\gamma_{i}\right)}^{2}} & \text{if}\;\max_{j}V\left(N_{i}\right)>B\left(N_{i}\right)\\ \frac{B\left(N_{i}\right)}{{\left(1+\gamma_{i}\right)}^{2}} & \text{else} \end{array}$$

being *j* the subset of nodes for which there exists an edge *E_i,j_* connecting node *N_i_* with node *N_j_* and being *i* < *j*, or in other words, each *N_j_* is a direct child node of *N_i_*.

However, this equation does not take into account the possible presence of other searchers in the current area of the root node. Thus, in order to consider this issue, some changes have to be made in order to correct the first level node values. In this sense, the nodes that are far away from the other searchers in that area and close to the current agent increase their values as follows:

(7) $$V^{\prime }\left(N_{b}\right)=\frac{V\left(N_{b}\right)}{d^{\gamma 0}\left(c_{0},c_{b}\right)}\prod_{r=0}^{\gamma 0}d\left(c_{0}^{r},c_{b}\right)$$

where the function *d*(*x, y*) measures the distance between cells *x* and *y*, $c_{0}^{r}$ is the position of the agent *r*, and *b* denotes the subset of first level nodes, that is, there exists and edge *E*_0,*b*_ connecting the root node *N*_0_ with *N_b_*.

Finally, the next goal *G* is decided as the position *c_b_* of the first level node *N_b_* that maximizes the corrected values:

(8) $$G=\arg\underset{c_{b}}{\max}V^{\prime }\left(N_{b}\right)$$

Since the best next child nodes has been saved during the evaluation process, a full set of way points can be obtained beginning in *G* until the last node of the path marked in this branch. A simple navigation function follows each one of these way points. When the final planned node is reached the searcher plans again building a new tree from its new position.

## 5. Experiments and Results

### 5.1. Dynamic Targets

The targets of the search could consist in a vast range of agents of distinct types. For instance, they could be a group of humans, animals, or a team of mobile robots. As we said, we are interested in modelling applications as, for instance, robots with human-robot interaction capabilities that are looking for humans in their proximities in order to interact with, or the case of seeking for malfunctioning robots that can be moving around without control. In this sense, for simulation purposes, it can be assumed that a target is some kind of abstract dynamic agent that makes movements to random destinations.

Consequently, in the simulations, for each target agent it has been assumed that it perfectly knows the environment and its localization in the map. In addition, the movement of the targets is limited in speed, and inside an environment of delimited dimensions.

Therefore, the movement of the targets has been implemented as follows. Each agent that acts as a dynamic target knows a discrete representation of the environment that describes the position of the obstacles in a grid. With this representation of the environment, they are able to select a random cell as destination. The A* algorithm [25] is used to plan a path to reach that cell. The target agent follows that path until it reaches its destination. Then, it selects a new random destination cell and the same process is repeated. The target only stops moving when it is detected by a searcher. When all the targets have been detected by the searchers the search is considered to be concluded.

### 5.2. Search Team

The team of searchers consists of a set of mobile agents that are able to sweep the environment detecting targets. Nowadays, there are mobile robots that include sensors such as laser range finders or omnidirectional cameras that allow them to sense the environment within a range around them. From laser scans and/or from omnidirectional images, there exists classifiers that are able to identify different kind of objects. For these reasons, it is assumed that each searcher is able to detect mobile targets within a determined area around it.

Regarding the knowledge of the environment, two cases have been studied in this paper:

- The searchers have full knowledge of the map of the environment: This case represents the situation in which the team of searchers needs to find and detect a group of mobile agents inside a known environment, as for instance inside a previously mapped building.
- The searchers have not a priori knowledge of the environment and build cooperatively a map of it: This case tries to model the hypothetical situation in which the searchers have to find a group of dynamic agents in a completely unknown environment.

As in the case of the dynamic targets, in both cases the map consists in a discrete representation of the environment that describes the position of the obstacles in a grid. When the map is not a priori known, cells can be also labelled as unknown besides free or occupied. A ray tracing function with the information of the laser scans clears or marks the cells accordingly using a reflection probability model [26]. Furthermore, the localization of the searchers is assumed to be known. There exists several techniques to provide localization for a team of mobile robots, as for instance, a GPS device in outdoor environments, Monte Carlo localization [27], when the map is known, or Simultaneous Localization and Mapping (SLAM) algorithms [28] to build the map and localize the robots concurrently. Since this is not the focus of this paper, the localization is assumed to be known and its error is considered to be not significant. In this way, the localization and the map (partial or complete) is shared by all the searchers. However, in contrast to other authors [1], the position of the targets remains unknown to them.

As it was explained in Section 3, a map that stores the probability of finding a target in each cell of the grid is built by the GBBF. This map can be understood as a generalization of an occupancy grid map where each cell stores the probability of being occupied by a target. This map is constructed in a centralized way and is also shared by all the searchers. Then, using the information from this map, the searchers apply the search algorithm exposed in Section 4 in order to find a route to the areas where it is more likely to find a target.

### 5.3. Simulator Description

**Figure 3.**
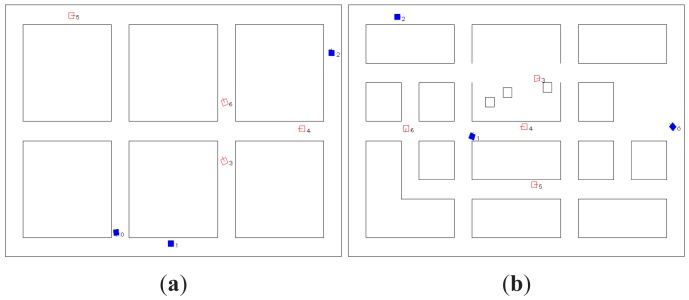
Simulation scenarios. The searchers are shown as filled squares and the dynamic targets as empty squares. (**a**) Scenario 1; (**b**) Scenario 2.

The method proposed in this paper has been tested in a simulation software developed for this purpose. Figure 3 shows the two scenarios included in this simulator that were used during the tests. Both scenarios have fixed dimensions of 38 × 26 *m*. All the maps are built with a resolution of 0.1 *m*.

The simulated agents, both searchers and dynamic targets, move with a linear speed limited to 0.3 *m/s* and the angular speed limited to 0.25 *rad/s*. The sensors of the pursuers have an omnidirectional range of 6 *m*. The experiments were carried out using a simulation time with a fixed time period of 0.33 *s*, which means that independently of the time needed for the calculations we assume that the elapsed time between data acquisition and the new commands given to the agents in order to go to next waypoint according to the planning is fixed.

The GBBF has a complexity *O*(*T*) with the number of cells *T*. Therefore, the algorithm is mainly limited with the size of the area to explore. The complexity of the search planning algorithm is dependent of the number of nodes *N* found in the process of creation of the tree. This depends on the structure of the environment. In this sense, it is necessary to process each node in order to find its associated region *R_i_*. Furthermore, for each node added to the tree, it is required to find the node with the minimum cost in the remaining unprocessed nodes list in order to continue building the tree. This list can be maximum *N* − 1 in size. Thus, the complexity is *O*(*N*(*R* + *N* − 1)), being *R* the maximum number of cells for a node according to the sensor maximum range. Typically, *R* is fixed and much bigger than *N* making the complexity almost linear with number of nodes *N*. However, the planning does not take place all the time, only when the last planning has finished. Consequently, the computation of the search tree is not critical and the main bottleneck is the size of the map. The size of the scenarios chosen for simulation allows a fast update in real time.

The experiments were performed for each scenario varying the number of searchers and targets in a range of 1–4 for searchers and 1–10 for targets. Each experiment was performed 25 times randomly changing the initial positions of the agents. All the results presented hereafter are the average of all these simulations.

Next, we analyse the results in terms of search time for known and unknown environments.

### 5.4. Experiments in Known Environments

**Figure 4.**
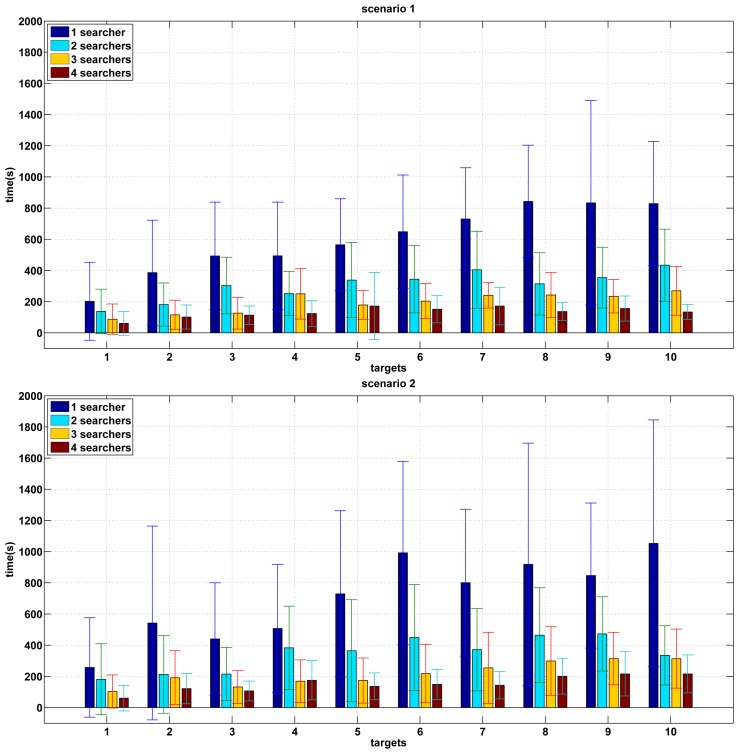
Results of experiments with a known map.

Figure 4 shows the results of the experiments performed in known environments for the two proposed scenarios. It is shown the mean and standard deviation of the experiments carried out for a range of 1–4 in the number of searchers and 1–10 in the number of targets. In this case, it is assumed that the searchers know the occupancy map of the scenario where the search is going to be carried out from the beginning of the search. Thus, they do not need to create an occupancy map. When there is only one searcher, the search time grows almost linearly when the number of targets is low. When the number of targets is high the total time remains almost constant. That can be observed in both scenarios. This is because only one searcher has to explore an extensive environment.

As the number of searchers grows, the search time decreases for a given number of targets. Since the size of the map is constant, it is reasonable to assume that, as the number of searchers increases, the search time is reduced. The search time is inversely proportional to the number of robots approximately.

The most complex scenarios with more loops, like Scenario 2, require a higher search time. This is observed for all the performed tests and it is due to the higher number of escape ways. In this case, the variability in the required time for a single robot is greater than in Scenario 1.

From a given number of searchers (approximately 3 or more for the environments in the simulations) and for a given number of targets (about 6 in Scenario 1 and 8 in Scenario 2), as the number of targets grows, the search time does not grow significantly. This can be justified because the environment is covered in few steps. Thus all the targets are found quickly and independently of their number.

The standard deviation of the experiments that were carried out is high despite the fact of having performed 25 tests in every case. This is mainly observed in the case of one pursuer. This is a consequence of the initial positions being established randomly and also the paths of the targets being random.

### 5.5. Experiments in Unknown Environments

**Figure 5.**
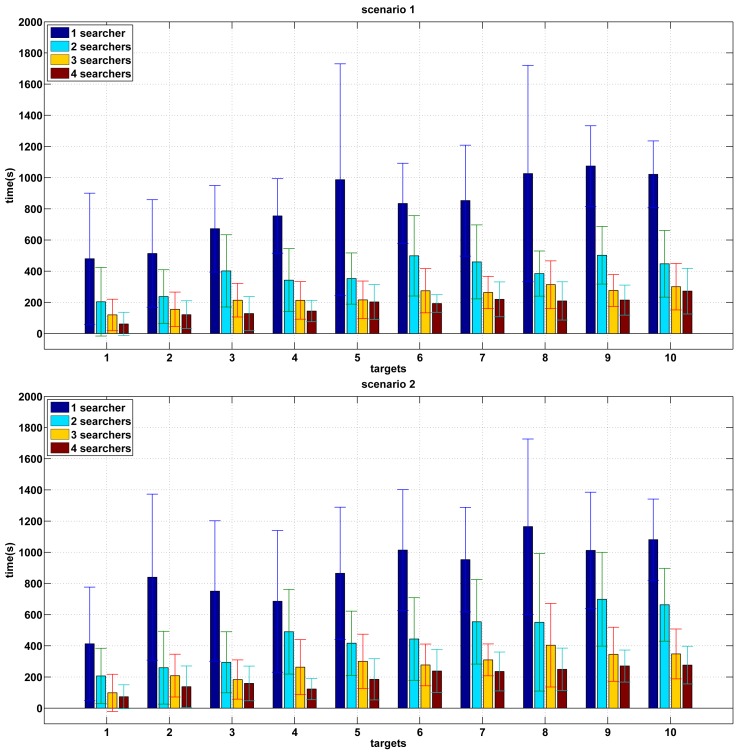
Results of experiments with an unknown map.

Figure 5 shows the results of the experiments performed in unknown environments. The two graphs show the mean and standard deviation of the experiments carried out for a range of 1–4 in the number of searchers and 1–10 in the number of targets in scenario 1 and 2 respectively. In these experiments, the searchers do not have a previous knowledge of the environment so they need to build a map of the environment while they are searching for targets.

The results are quite similar to the case of known maps. As the number of searchers grows, the search time decreases for a given number of targets. In this sense, the search time is approximately inversely proportional to the number of robots.

However, a considerable increase in the search time is observed for all combinations. The main cause of this delay is that the pursuers have no a priori knowledge of the environment. Thus the destination selection is not the best, since the pursuers do not have enough information about the environment.

There are fewer differences between both scenarios since there is no information available at the beginning in both cases.

## 6. Conclusions and Future Work

In this paper a new search and exploration algorithm that allows a team of robots to cooperatively look for a set of moving targets inside a delimited environment has been presented. An estimation of the areas of the environment that are more likely to hold a target has been obtained. The only assumption that we make about the movement of the targets is a random walk model and a top speed. In this sense, a technique to create a map of the probability for a cell to hold a target has been explained using a grid-based Bayesian Filter. Besides, a navigation algorithm has been designed in order to command the robots to the more likely areas to find a target including some multi-agent coordination mechanisms.

The algorithm has been successfully tested in known and unknown environments. In this sense, the algorithm has been demonstrated to work with or without previous knowledge of the environment and with no other information of the movement of the dynamic targets apart from their top speed. The results in unknown environments have been found to be reasonably efficient.

As future work, the use of this algorithm in real applications will be considered. Furthermore, the most complex case in which the target tries to deliberately avoid the searcher will be studied. In this sense, a subdivision of the environment using a cyclic graph instead of a tree can be useful to coordinate the pursuers in order to find cooperative strategies to locate all the evaders. Unsupervised learning incorporating semantic mapping information will be also studied in order to enhance the search.
